# Clinical outcomes of endoscopic resection for rectal neuroendocrine tumors: Advantages of endoscopic submucosal resection with a ligation device compared to conventional EMR and ESD

**DOI:** 10.1002/deo2.35

**Published:** 2021-09-01

**Authors:** Yuki Kamigaichi, Ken Yamashita, Shiro Oka, Hirosato Tamari, Yasutsugu Shimohara, Tomoyuki Nishimura, Katsuaki Inagaki, Yuki Okamoto, Hidenori Tanaka, Ryo Yuge, Yuji Urabe, Koji Arihiro, Shinji Tanaka

**Affiliations:** ^1^ Department of Endoscopy Hiroshima University Hospital Hiroshima Japan; ^2^ Department of Gastroenterology and Metabolism Hiroshima University Hospital Hiroshima Japan; ^3^ Division of Regeneration and Medicine Center for Translational and Clinical Research Hiroshima University Hospital Hiroshima Japan; ^4^ Department of Anatomical Pathology Hiroshima University Hospital Hiroshima Japan

**Keywords:** endoscopic mucosal resection, endoscopic submucosal dissection, endoscopic submucosal resection with a ligation device, rectal neuroendocrine tumor

## Abstract

**Objectives:**

There are some endoscopic resection (ER) methods for neuroendocrine tumors (NETs), however, which method is most useful remains unclear. This study aimed to compare the outcomes of different ER techniques, such as conventional endoscopic mucosal resection (cEMR), endoscopic submucosal dissection (ESD), and endoscopic submucosal resection with a ligation device (ESMR‐L) for rectal NETs.

**Methods:**

We retrospectively analyzed 96 consecutive patients with 102 rectal NETs of less than 10 mm in diameter who underwent ER between January 2001 and December 2019 at Hiroshima University Hospital. We compared the clinical outcomes of each ER method (cEMR 60 lesions, ESD 21 lesions, and ESMR‐L 21 lesions), divided according to the treatment periods, and evaluated the risk factors for vertical margin (VM) positivity in relation to clinicopathological and endoscopic characteristics.

**Results:**

As for the mean procedure time, ESD took significantly longer to perform than the other methods. The histological complete resection rate was 80% (48/60) for cEMR, 85.7% (18/21) for ESD, and 100% (21/21) for ESMR‐L, and the VM positive rate was 20% (12/60) for cEMR, 14.3% (3/21) for ESD, and 0% (0/21) for ESMR‐L, with no significant difference. However, the tumor‐front‐to‐VM distance was significantly longer in the ESMR‐L group than in the cEMR and ESD groups. cEMR and ESD were both significant risk factors for VM positivity. No perforation or local recurrence was observed in all methods.

**Conclusions:**

ESMR‐L is the most useful ER method for small rectal NETs.

## INTRODUCTION

The number of colorectal neuroendocrine tumors (NETs) successfully diagnosed, particularly rectal NETs, has been increasing due to the widespread use and quality improvement of colonoscopy techniques.^1^, ^2^ According to Japanese guidelines, metastasis‐related factors in rectal NETs include tumor size, depth, vascular invasion, cell proliferative potential (Ki‐67 index, number of mitotic figures), and the presence of superficial depressions and ulceration.^3^ Among these factors, tumor size is considered the most reliable factor prior to treatment for rectal NETs.^3^, ^4^, ^5^, ^6^, ^7^

There is an international consensus that rectal NETs of less than 10 mm in diameter are eligible for endoscopic resection (ER) because of their low lymph node metastasis (<2%), distant metastasis (<0.7%),^8^, ^9^ and good 5‐year survival rate (98.9–100%).^10^, ^11^, ^12^

Various ER methods are utilized for the resection of rectal NETs, including conventional endoscopic mucosal resection (cEMR), endoscopic submucosal dissection (ESD), and modified EMRs, such as pre‐cutting EMR, cap‐assisted EMR, and endoscopic submucosal resection with a ligation device (ESMR‐L). Previous reports demonstrated that ESMR‐L achieved a complete resection rate not inferior to ESD.^13^, ^14^, ^15^ However, ESD requires a longer procedure time and more advanced endoscopic techniques than ESMR‐L. Moreover, there are few reports comparing the results of different ER methods.

In this study, we compared the outcomes of cEMR, ESD, and ESMR‐L for rectal NETs of less than 10 mm in diameter. We also measured and compared the tumor‐front‐to‐vertical margin (VM) distance in the resected specimen for each ER method to assess the efficacy of histologically complete resection.

## METHODS

### Patients

**FIGURE 1:**
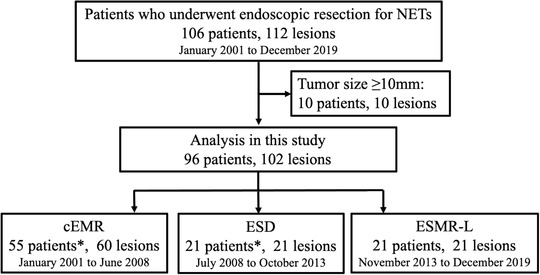
Patients enrolled in this study *Overlapped. Abbreviations: cEMR, conventional endoscopic mucosal resection; ESD, endoscopic submucosal dissection; ESMR‐L, endoscopic submucosal resection with a ligation device; NET, neuroendocrine tumor .

**FIGURE 2:**
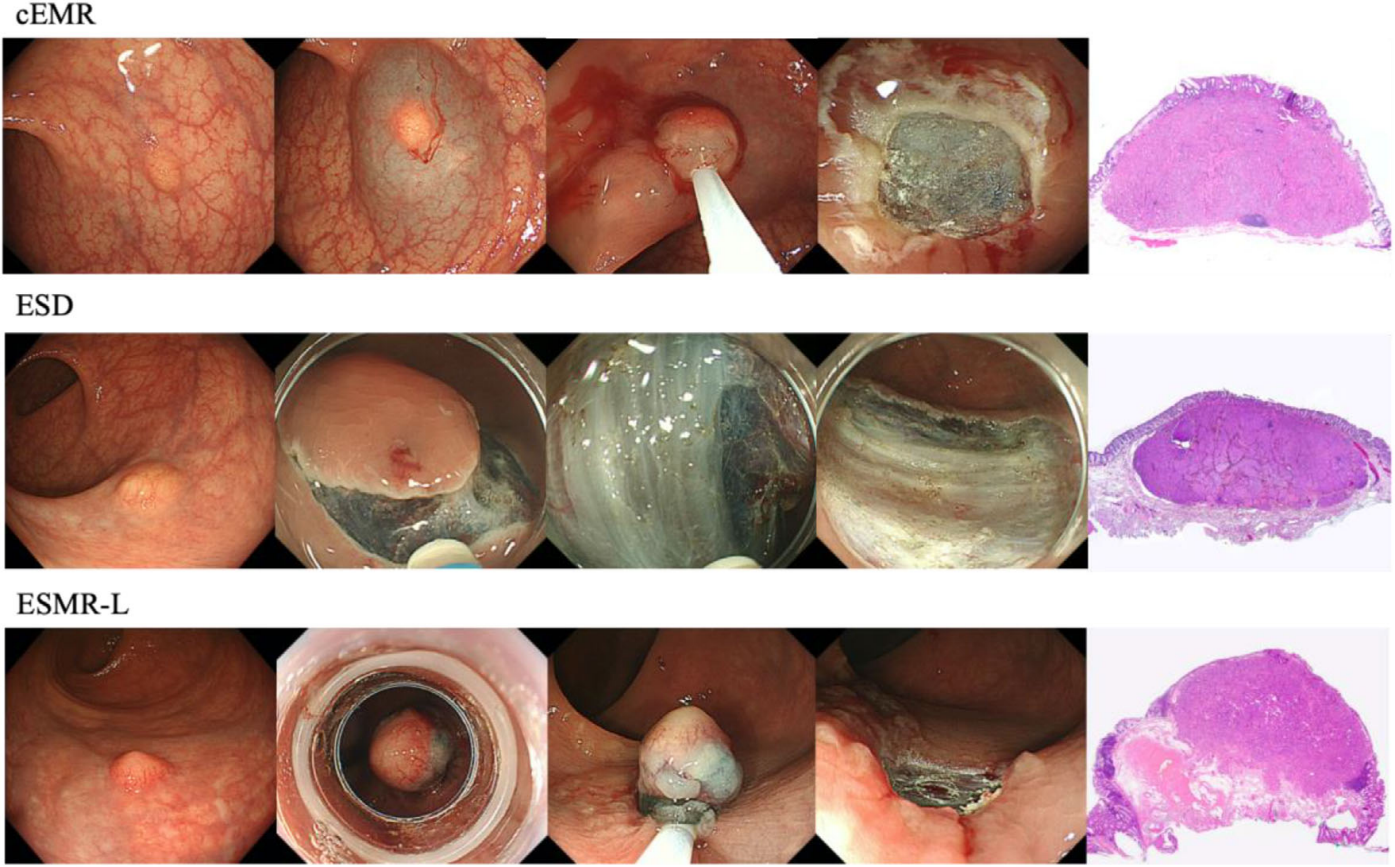
Each endoscopic resection procedures for rectal neuroendocrine tumors Abbreviations: cEMR, conventional endoscopic mucosal resection; ESD, endoscopic submucosal dissection; ESMR‐L, endoscopic submucosal resection with a ligation device

This study enrolled a total of 106 consecutive patients who underwent ER for 112 rectal NETs between January 2001 and December 2019 at Hiroshima University Hospital. Data related to patient and lesion characteristics, procedure outcomes, pathological results, and postoperative clinical course were recorded. We excluded cases with lesions of 10 mm or larger in diameter (10 patients, 10 lesions), and finally analyzed a total of 96 patients with 102 rectal NETs of less than 10 mm in diameter (**Figure** **1**, **2**). Patients were divided into three ER method groups according to the treatment period as follows: cEMR (55 patients, 60 lesions) from January 2001 to June 2008, ESD (21 patients, 21 lesions) from July 2008 to October 2013, and ESMR‐L (21 patients, 21 lesions) from November 2013 to December 2019.

### Indications of ER for rectal NETs

NETs were diagnosed via endoscopic or histopathological examination. The indications for ER for NETs were as follows: no obvious ulceration, tumors located within the submucosal layer, and no local or distant metastasis.^9^, ^16^, ^17^, ^18^ All patients underwent endoscopic ultrasonography (UM‐DP20‐25R or UM‐DP12‐25R; Olympus, Tokyo, Japan) before ER to determine the depth of invasion. Computed tomography (CT) was also performed to confirm the presence of metastases before ER.

### Endoscopic procedures

Figure 1 shows the procedure for each ER method. ER was conducted by experienced endoscopists. cEMR was performed using a single‐channel colonoscope (PCF‐Q260AZI or PCF‐H290ZI; Olympus). After injecting 10% glycerin solution mixed with a small amount of indigo‐carmine to lift the lesion, the base of the lesion was captured with a snare (SnareMaster; Olympus) and cut using an electrosurgical current. ESD was performed using a single‐channel colonoscope (PCF‐Q260AZI or PCF‐H290TI; Olympus) with DualKnife or DualKnife‐J (Olympus). After injecting 10% glycerin solution and/or 0.4% sodium hyaluronate solution (MucoUp; Johnson & Johnson, New Brunswick, NJ, USA) mixed with a small amount of indigo carmine into the submucosal layer, a circumferential incision was made, and submucosal dissection was then performed. ESMR‐L was conducted using a ligation device (Sumitomo Bakelite Co. Ltd., Tokyo, Japan) attached to a single‐channel colonoscope (PCF‐Q260AZI or PCF‐H290ZI). After injecting 10% glycerin solution mixed with a small amount of indigo carmine into the submucosal layer, the lifted lesion was suctioned into the ligation device, and an elastic band was deployed.^19^ The lesion was resected by snaring under the band.

### Pathological diagnosis

The resected specimens were stretched, pinned on the flat styrofoam board, fixed with 10% formalin solution, and then evaluated under a microscope. Histological diagnoses, such as histopathological type, World Health Organization (WHO) grade, depth of invasion, horizontal margin (HM) or VM, and lymphovascular invasion were evaluated. VM positivity was defined as the presence of tumor invasion at the vertical resection margin. Lymphovascular invasion was evaluated by hematoxylin and eosin staining. However, depending on the situation, immunostaining (Victoria blue, Elastica van Gieson, D2‐40) was performed, as a reference for diagnosis. Immunohistochemical staining for neuron‐specific enolase, synaptophysin, and chromogranin A was also performed to confirm the diagnosis. Ki‐67 was used to assess tumor proliferation and classify the WHO grade of the tumors based on 2019 WHO classification of tumors of the digestive system.^20^ We also measured the tumor‐front‐to‐VM distance in the resected specimen. In cases with VM positivity, this distance was assumed to be zero. All pathological specimens were evaluated by an expert pathologist (K. A.).

### Evaluation

We evaluated the clinicopathological characteristics, such as age, sex, tumor size, tumor location, and clinical outcomes of NETs, including en bloc resection rate, histologically complete resection rate, procedure time, procedure‐related complications (delayed bleeding and perforation), pathological diagnosis, and existence of recurrence for each ER method to compare their efficacy and safety. We also evaluated the risk factors for VM positivity using the following variables: sex, age, tumor size, tumor location, presence of depression, ER method, en bloc resection rate, and pathological diagnosis (lymphovascular invasion and WHO grade). En bloc resection was defined as the tumor being entirely endoscopically resected in one piece. Histologically, complete resection was defined as en bloc resection without positive HM or VM of the resected specimen. The patients underwent colonoscopy and CT at 6 and 12 months after ER, and every year thereafter, to determine the presence of local recurrence and metastasis.

This study was performed in accordance with the principles of the Declaration of Helsinki in compliance with good clinical practice guidelines and local regulations. The study was approved by the Institutional Review Board Ethics Committee of the Hiroshima University Hospital (No. E‐1637).

### Statistical analysis

The data are presented as mean ± standard deviation (SD) or median (range). Pearson's chi‐square test, Fisher's exact test, Student's *t*‐test, and the Wilcoxon rank‐sum test with Holm–Bonferroni method were used to compare the qualitative and quantitative variables. Statistical significance was set at *p* < 0.05.

All statistical analyses were performed using the JMP software version 14 (SAS Institute, Inc., Cary, NC, USA).

## RESULTS

### Baseline characteristics

Table 1 shows the clinicopathological characteristics of the 96 patients (male/female: 58/38, average age: 56.3 years) with 102 lesions. The lymphatic invasion rate was 1.7% (1/60) for cEMR, 23.8% (5/21) for ESD, and 14.3% (3/21) for ESMR‐L, which was significantly higher for ESD than for cEMR (*p* < 0.01). There were no significant differences in sex, age, tumor size, tumor location, presence of depression, or other pathological diagnoses (venous invasion and WHO grade) among the three groups.

**TABLE 1:** Baseline characteristics of tumors (*N* = 102)

		**Resection method**			
**Variables**	**Total*N* = 102**	**cEMR*n* = 60**	**ESD*n* = 21**	**ESMR‐L*n* = 21**	***p‐*value**
Sex, male	62 (60.8)	40 (66.7)	11 (52.4)	11 (52.4)	NS
Age, mean ± SD, years old	56.3 ± 12.7	56.4 ± 12.9	56.0 ± 11.4	56.1 ± 13.7	NS
Tumor size, mean ± SD (mm)	5.1 ± 1.6	4.9 ± 1.6	5.8 ± 1.4	5.1 ± 1.6	NS
Location					
Ra	21 (20.6)	12 (20.0)	5 (23.8)	4 (19.0)	NS
Rb	81 (79.4)	48 (80.0)	16 (76.2)	17 (81.0)	NS
Presence of depression	8 (7.8)	4 (6.7)	4 (19.0)	0 (0)	NS
Pathological diagnosis					
Lymphatic invasion (+)	9 (8.8)	1 (1.7)	5 (23.8)	3 (14.3)	EMR vs. ESD∗
Venous invasion (+)	10 (9.8)	4 (6.7)	4 (19.0)	2 (9.5)	NS
WHO grade					
Grade 1	96 (94.1)	57 (94.9)	19 (90.5)	20 (95.2)	NS
Grade 2	6 (5.9)	3 (5.1)	2 (9.5)	1 (4.8)	NS
					(%)

Abbreviations: cEMR, conventional endoscopic mucosal resection; ESD, endoscopic submucosal dissection; ESMR‐L, endoscopic submucosal resection with a ligation device; NS, not statistically significant; Ra, rectum above the peritoneal reflection; Rb, rectum below the peritoneal reflection; WHO, World Health Organization.

*p < 0.01, **p < 0.05.

### Clinical outcomes according to the resection method

Clinical outcomes of each ER method are presented in Table 2. The procedure time for ESD (13.5 ± 3.1 min) was significantly longer than that for cEMR (3.3 ± 0.8 min) and ESMR‐L (5.7 ± 1.2 min) (*p* < 0.01). En bloc and histologically complete resection rates were 100% (102/102) and 85.3% (87/102), respectively. Histologically complete resection rate in each ER method was 80% (48/60) for cEMR, 85.7% (18/21) for ESD, and 100% (21/21) for ESMR‐L. The VM positive rate was 20% (12/60) for cEMR, 14.3% (3/21) for ESD, and 0% (0/21) for ESMR‐L, with no significant difference. However, the tumor‐front‐to‐VM distance in ESMR‐L (641.5 ± 763.8 μm) was significantly longer than that in cEMR (188.9 ± 199.1 μm) and ESD (202.8 ± 125.4 μm) (*p* < 0.05). There were no significant differences in complications among the three groups. No local recurrence was observed during the follow‐up period (mean, 53.8 months; range, 6–156 months).

**TABLE 2:** Clinical outcomes according to each resection method (*N* = 102)

		Resection method			
Variables	Total*N* = 102	cEMRn = 60	ESDn = 21	ESMR‐Ln = 21	*p‐*value
Procedure time, mean ± SD, min	4.7 ± 1.6	3.3 ± 0.8	13.5 ± 3.1	5.7 ± 1.2	cEMR vs. ESD^*^, ESD vs. ESMR‐L^*^
En bloc resection	102 (100)	60 (100)	21 (100)	21 (100)	NS
Histologically complete resection	87 (85.3)	48 (80.0)	18 (85.7)	21 (100)	NS
Horizontal margin (+)	0 (0)	0 (0)	0 (0)	0 (0)	NS
Vertical margin (+)	15 (14.7)	12 (20.0)	3 (14.3)	0 (0)	NS
Tumor‐front‐to‐vertical‐margin distance (μm)	309.6 ± 468.3	188.9 ± 199.1	202.8 ± 125.4	641.5 ± 763.8	cEMR vs. ESMR‐L^**^, ESD vs. ESMR‐L^**^
Complication					
Delayed bleeding	11 (10.8)	7 (11.7)	3 (14.3)	1 (4.8)	NS
Perforation	0 (0)	0 (0)	0 (0)	0 (0)	NS
Local recurrence	0 (0)	0 (0)	0 (0)	0 (0)	NS
					(%)

Abbreviations: cEMR, conventional endoscopic mucosal resection; ESD, endoscopic submucosal dissection; ESMR‐L, endoscopic submucosal resection with a ligation device; NS, not statistically significant.

*p < 0.01, **p < 0.05.

### Risk factors for VM positivity

We analyzed the risk factors for VM positivity (Table 3). Of the 102 tumors (96 patients), 15 (14.7%) were VM positive. There were no significant differences in sex, age, tumor size, location, presence of depression, or pathological diagnosis between the two groups with or without VM positivity. In addition, cEMR and ESD were identified as the only significant factors associated with VM positivity (*p* < 0.05).

**TABLE 3:** Risk factor for vertical margin positivity (*N* = 102)

	**Vertical margin**		
**Variables**	**(+)*n* = 15**	**(‐)*n* = 87**	***p‐*value**
Sex, male	9 (60.0)	53 (60.9)	NS
Age, mean ± SD, years old	59.2 ± 11.3	55.8 ± 12.9	NS
Tumor size, mean±SD (mm)	5.1 ± 1.4	5.1 ± 1.6	NS
Location			
Ra	5 (33.3)	16 (18.4)	NS
Rb	10 (66.7)	71 (81.6)	NS
Presence of depression	2 (13.3)	6 (6.9)	NS
Resection method			
cEMR	12 (20.0)	48 (80.0)	NS
ESD	3 (14.3)	18 (85.7)	NS
ESMR‐L	0 (0)	21 (100)	<0.05
En bloc resection	15 (100)	87 (100)	NS
Pathological diagnosis			
Lymphatic invasion (+)	0 (0)	9 (10.3)	NS
Venous invasion (+)	2 (13.3)	8 (9.2)	NS
WHO grade			
Grade 1	14 (93.3)	82 (94.3)	NS
Grade 2	1 (6.7)	5 (5.7)	NS
			(%)

Abbreviations: cEMR, conventional endoscopic mucosal resection; ESD, endoscopic submucosal dissection; ESMR‐L, endoscopic submucosal resection with a ligation device; NS, not statistically significant; Ra, rectum above the peritoneal reflection; Rb, rectum below the peritoneal reflection; WHO, World Health Organization.

Among the 15 patients who were VM positive, 12 patients were followed up endoscopically upon their own request, one patient underwent additional surgery, and two patients were lost to follow‐up. All patients who were followed up had no local recurrence or metastasis during the follow‐up period (mean: 72.9 months, range: 6–156 months).

## DISCUSSION

Our study revealed that ESMR‐L is the simplest and most reliable ER method for rectal NETs of less than 10 mm in diameter. Histologically, the complete resection rate of ESMR‐L is 100%, which was equally impressive as the results shown in several previous studies (93.3‐100%).^10^, ^21^, ^22^, ^23^, ^24^ The rate of cEMR was 80% and that of ESD was 85.7%, which was also not significantly different from previous reports (50‐77.4% in cEMR,^23^, ^25^, ^26^, ^27^, ^28^ 75–97.7% in ESD^13^, ^23^, ^25^, ^29^) and was lower than that of ESMR‐L. Lee et al.^24^ reported that modified EMR, including ESMR‐L, precutting EMR, and strip biopsy, had a significantly higher histologically complete resection rate than conventional EMR. Kim et al.^23^ compared conventional EMR, ESMR‐L, and ESD for NETs less than 10 mm in diameter and reported that ESD/ESMR‐L had a significantly higher histologically complete resection rate than conventional EMR. Ebi et al.^25^ also showed that resection by ESMR‐L, cap‐assisted EMR, and ESD had a higher complete resection rate than conventional EMR. In our institution, we selected the ER method according to each period. Especially, in 2013, we introduced ESMR‐L because of its simple and reliable procedures regardless of operators’ skill. One of the most significant strengths of our study was that there was no selection bias.

One of the most important reasons for the superior outcomes of ESMR‐L is its low VM positive rate. Previous reports revealed that the VM positive rate was 22.6–50% in cEMR,^23^, ^25^, ^26^, ^27^, ^28^ 0–29.2% in ESD,^13^, ^23^, ^25^, ^29^ and 0–10% in ESMR‐L.^13^, ^23^, ^25^, ^26^, ^28^, ^29^

ESMR‐L was performed to resect the submucosal layer by suction using a ligature device to ensure a safe margin. After the submucosa was sufficiently suctioned into the transparent cap, a ligature band was placed, and the snare was resected just below the band; this theoretically provides a deeper and wider vertical resection margin.^30^ Better VM due to band ligation may have contributed to the low VM positive rate. In this study, a comparison of the tumor‐front‐to‐VM distance among all ER methods showed that ESMR‐L had the maximal VM significantly. There are few previous studies on VM distance. Lim et al.^15^ reported that the lateral and VM were longer in the ESMR‐L group than in the ESD group. Kim et al.^23^ showed that the distance from the tumor front to the VM was significantly greater in the ESMR‐L and ESD groups than in the EMR group.

No perforation occurred in any of the cases in this study. In a previous report, 0–2.5% (one case) of perforation was reported with ESMR‐L, indicating that it is safe and can be treated using ER methods.^13^, ^23^, ^25^, ^26^, ^27^, ^28^, ^29^

In the analysis of VM positivity, the only significant risk factor was the non‐selection of ESMR‐L as an ER method. Various experienced endoscopists were able to achieve a high histologically complete resection rate of ESMR‐L, even if they were not familiar with the procedure. Therefore, ESMR‐L could be the first choice as an ER method for small NETs, regardless of the endoscopist's experience. In performing ESD, it is essential to pay attention to the aforementioned points and make an effort to dissect just above the muscle layer with a clear field of view.

After ER, there were two major management courses: follow‐up with or without additional treatment, including surgery. The decision to select either was based on the pathological diagnosis, particularly the presence of risk factors for lymph node metastasis, of rectal NETs resected by ER. Additional surgery was recommended for cases judged to be at high risk of metastasis; however, there was insufficient consensus on its criteria.^31^ In this study, the rate of lymphatic invasion was 8.8%, and the rate of venous invasion was 9.8%. Additional surgery was performed in 22.2% (2/9) of cases with lymphatic invasion and 20% (2/10) with venous invasion, while other cases were followed up mainly at the request of the patients.

Among the 15 VM positive cases, two cases in the cEMR group and one case in the ESD group underwent additional surgery. Currently, there is no consensus on surveillance after ER for NETs. Some guidelines recommend surveillance by colonoscopy and CT every 12 months after local resection, while others state that there is no justification for recommending such surveillance.^4^, ^9^, ^16^, ^32^ However, previous reports indicated that the recurrence period was 5–12 years.^33^ Thus, a common view was expressed that further long‐term surveillance is necessary for patients who underwent ER for NETs due to its slow growth.^3^, ^4^, ^9^, ^34^

This study has some limitations. First, this was a retrospective study conducted in a single center and the sample size was relatively small. Second, although ER methods were selected according to each period without selection bias, the development of treatment strategies and devices may have influenced the results. Third, each ER method was selected for each treatment period and was not conducted by precisely uniform operator's endoscopic experience. Fourth, immunostaining was not performed for all cases, resulting in a bias in the rate of lymphovascular invasion by ER methods. Finally, the surveillance period is not enough to analyze the long‐term outcome, including prognosis and recurrence.

In conclusion, in the technical aspect, ESMR‐L is considered to be the simplest and most reliable ER method for rectal NETs of less than 10 mm in diameter.

## CONFLICT OF INTEREST

The authors have no conflict of interest to declare.

## FUNDING INFORMATION

None.
